# Clinical Significance of Cardio-Ankle Vascular Index in Stage A/B Heart Failure Patients With Type 2 Diabetes Mellitus

**DOI:** 10.14740/jocmr6680

**Published:** 2026-08-26

**Authors:** Takashi Hitsumoto

**Affiliations:** Hitsumoto Medical Clinic, 2-7-7, Takezakicyo, Shimonoseki City, Yamaguchi 750-0025, Japan. Email: thitsu@jcom.home.ne.jp

**Keywords:** Cardio-ankle vascular index, Stage A/B heart failure, Type 2 diabetes mellitus, Cardiac function marker, Skin autofluorescence, Sarcopenia, Glucagon-like peptide-1 receptor agonists, Brain natriuretic peptide

## Abstract

**Background:**

Heart failure (HF) is a medical condition with a poor prognosis; therefore, it is important to consider treatment strategies from the pre-onset stage, i.e., at stage A/B HF. However, in addition to type 2 diabetes mellitus (T2DM) being a risk factor for HF, it has also been reported that the prognosis of HF is worse among patients with T2DM than among those without it. This cross-sectional study aimed to clarify the clinical significance of the cardio-ankle vascular index (CAVI), an indicator of arterial stiffness, in stage A/B HF patients with T2DM.

**Methods:**

In total, 292 stage A/B HF patients (113 men and 179 women) with T2DM were enrolled, and the relationships between CAVI and various clinical parameters were evaluated. Furthermore, cut-off values of CAVI and brain natriuretic peptide (BNP) for distinguishing between stage A HF and stage B HF were determined using a receiver operating characteristic curve, and these two markers were employed in combination to distinguish stage A HF from stage B HF.

**Results:**

CAVI was significantly associated with various parameters in this study. Among them, multiple regression analyses indicated that 11 explanatory factors (calf circumference, renin-angiotensin system inhibitors use, E/e' ratio, skin autofluorescence, subendocardial viability ratio, glucagon-like peptide-1 receptor agonists use, age, decreased estimated glomerular filtration rate, coronary artery disease, stage B HF, and sodium-glucose cotransporter 2 inhibitors use) were selected as independent variables for CAVI as the dependent variable (R^2^ = 0.663). However, CAVI and BNP thresholds for distinguishing stage A HF from stage B HF using the receiver operating characteristic curve were 9.0 and 34.0 pg/mL, respectively. In addition, compared to patients with low CAVI (≤ 9.0) and low BNP (≤ 34.0 pg/mL), those with high levels of both factors (CAVI > 9.0, BNP > 34.0 pg/mL) showed a 9.72-fold higher odds ratio (95% confidence interval: 3.24–19.92, P < 0.001).

**Conclusion:**

CAVI is significantly associated with early-stage HF in T2DM patients. Combining CAVI and BNP may provide a useful screening tool for subclinical HF risk stratification, although further prospective studies are required to elucidate causal relationships.

## Introduction

Several epidemiological studies have demonstrated the year-to-year increase in the incidence of heart failure (HF). HF is a major disease that modern cardiovascular medicine must address, not only from the perspectives of healthy life expectancy and health economics [1, 2]. Patients who have developed HF once reportedly experience frequent subsequent readmissions, a tendency observed in clinical practice [3]. Therefore, treatment strategies should be considered from the pre-symptomatic stage, i.e., A/B stage HF. However, type 2 diabetes mellitus (T2DM), besides contributing to the development of HF, is also associated with a worse prognosis of the latter in patients with T2DM than in those without it through the pathway of advanced glycation end products (AGEs), macrovascular ischemia, myocardial metabolic abnormalities, cardiac autonomic dysfunction, and volume load associated with the cardio-renal interaction [4, 5]. Meanwhile, several studies have reported the clinical importance of several blood biomarkers, including brain natriuretic peptide (BNP), as indicators of HF management and prediction [6–8]. However, these biomarkers alone do not solve all the problems plaguing HF management, and it is considered clinically significant to discover novel indicators that can help prevent HF from different perspectives. The cardio-ankle vascular index (CAVI) was developed as a novel physiological marker of arterial stiffness independent of blood pressure at the time of measurement [9]. Existing reports have examined the clinical significance of CAVI in patients with T2DM from the viewpoints of various pathogeneses and medical interventions [10–12]. Several researchers have pointed out the importance of CAVI as a predictor of cardiovascular disease (CVD) events in patients with cardiovascular risk factors, including T2DM [13–16]. Furthermore, there are several reports on the significance of CAVI in subclinical HF [17, 18]. However, there are still several unsolved points on the clinical significance of CAVI in patients with stage A/B HF with T2DM. Therefore, this cross-sectional study aimed to clarify the clinical significance of CAVI in stage A/B HF patients with T2DM.

## Materials and Methods

### Patients

Between February 2024 and January 2026, 292 consecutive patients with T2DM who visited Hitsumoto Medical Clinic with no symptoms of HF, and/or no history of hospitalization for HF, and for whom the clinical parameters necessary for this study, including CAVI, could be measured, were enrolled. The participants of this study were 113 men (39%) and 179 women (61%) with an average age of 70 years. Stage A was defined as patients with T2DM at risk for HF but without structural or functional cardiac abnormalities. Stage B was defined as asymptomatic patients with T2DM who had structural heart disease (e.g., documented coronary artery disease (CAD), valvular heart disease, or echocardiographic evidence of left ventricular hypertrophy) but no HF symptoms. Baseline numerical values of plasma BNP or specific continuous echocardiographic indices were not used as classification thresholds for group assignment [19]. This study was reported in accordance with the Strengthening the Reporting of Observational Studies in Epidemiology (STROBE) statement.

### Ethical considerations

This study was conducted in accordance with the Declaration of Helsinki. Informed consent was obtained from all study participants, and the study protocol was approved by the Institutional Review Board of Hitsumoto Medical Clinic (Date of Approval: January 9, 2024; Approval No. HMC-2024-1R-1).

### CAVI measurement

CAVI was measured using a VaSera CAVI device (Fukuda Electronics, Tokyo, Japan) based on existing reports [9, 20]. All CAVI measurements in this study were performed exclusively in the morning. The measurement method was as follows: First, the patient was placed in a supine position and allowed to rest for 10 min, after which the systemic blood pressure and pulse pressure were measured simultaneously. Pulse waves in the upper arm and ankle were measured using an inflatable cuff maintained at a pressure of 30–50 mm Hg. The CAVI measurement formula is as follows: CAVI = a(2ρ/ΔP) × ln (Ps/Pd) × (pulse wave velocity)^2^} + b, where a and b are constants, ρ is the blood density, ΔP is Ps−Pd, Ps is the systolic blood pressure, and Pd is the diastolic blood pressure. Patients who had atrial fibrillation at the time CAVI was measured, and those with an ankle brachial pressure index value of < 0.9 (suggestive of arteriosclerosis obliterans), were excluded from the study because of accuracy of CAVI. However, the mean coefficient of variation of CAVI measurements was less than 5%, indicating that there were no problems with CAVI reproducibility [9].

### Clinical parameter evaluation

In addition to CAVI, various clinical parameters such as body mass index, smoking habits, CAD, systolic blood pressure, serum lipid levels (serum low-density lipoprotein levels, serum high-density lipoprotein levels), echocardiographic findings, glucose-related parameters such as fasting blood glucose levels, hemoglobin A1c levels, and AGEs, serum lipid levels (serum low density lipoprotein cholesterol, serum high density lipoprotein cholesterol), BNP, estimated glomerular filtration rate (eGFR), high-sensitivity C-reactive protein (hs-CRP), the subendocardial viability ratio (SEVR), and medication status were evaluated. CAD was defined as the presence of angina pectoris and/or myocardial infarction managed either with coronary revascularization and/or oral medication. Echocardiography was performed using a commercial device (Philips Affiniti 30, Philips Healthcare, Bothell, WA, USA), with evaluation points of the imaging test comprising parameters such as left ventricular wall thickness, left ventricular extended period diameter, left ventricular ejection fraction (LVEF), left atrial dimension, and E/e' ratio, as a marker of left ventricular diastolic function. The BNP level was measured using a commercial kit (SHIONOSPOT Reader; Shionogi & Co., Osaka, Japan). Skin autofluorescence as a marker of AGEs *in vivo* was evaluated using a commercial device (AGE reader mu; DiagnOptics, Groningen, the Netherlands). The eGFR as a marker of kidney function was calculated using the Japanese criteria [21]. The SEVR was measured using the applanation arterial tonometry device (SphygmoCor, AtCor Medical, Australia). Regarding medications, the use of renin–angiotensin system (RAS) inhibitors, β-blockers, and statins in addition to anti-diabetes medications was investigated. Furthermore, patients receiving angiotensin receptor-neprilysin inhibitors (ARNIs) were not included in this study.

### Statistical analysis

**Figure 1:**
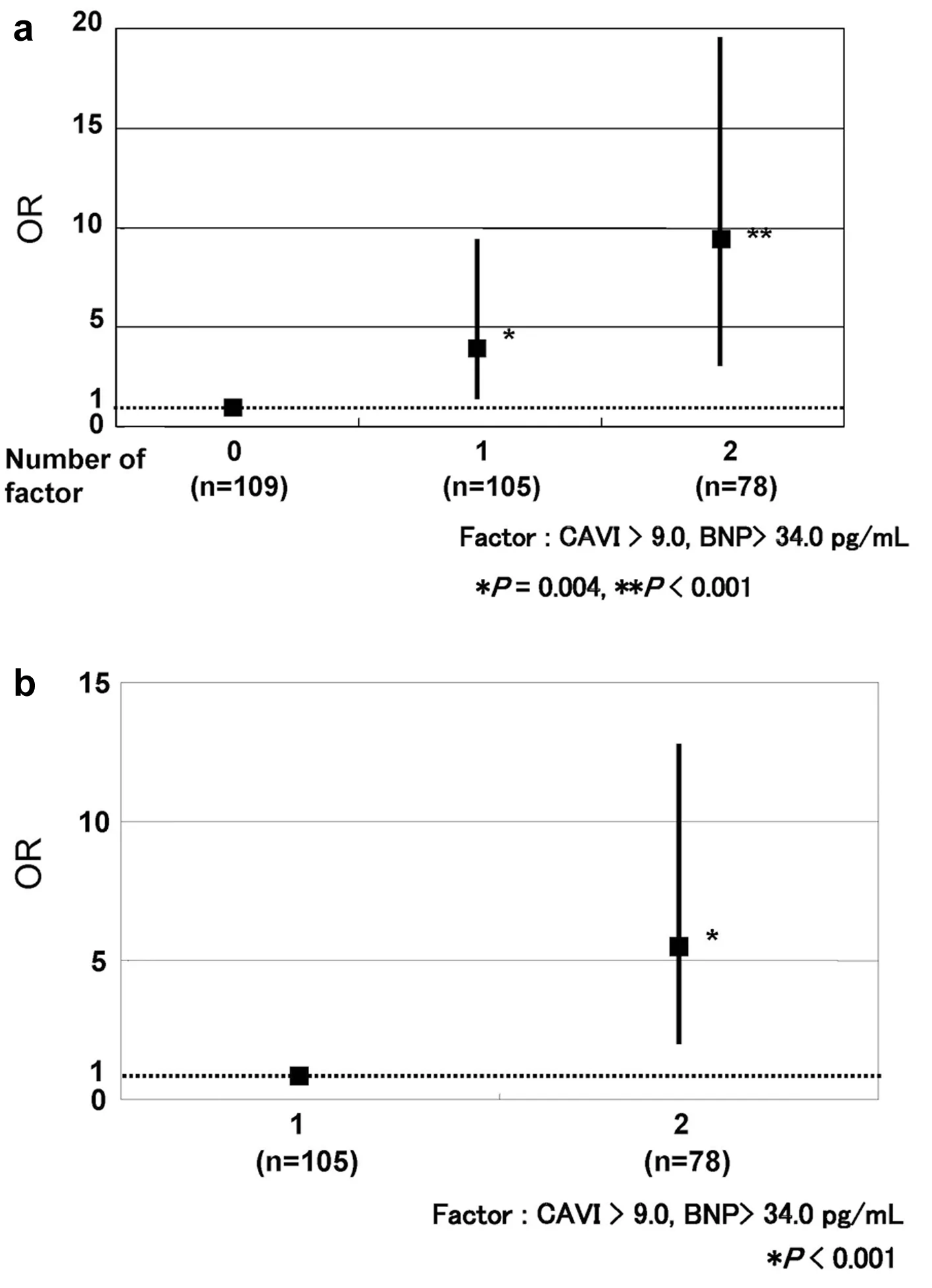
Association between the combination of high CAVI and high BNP and the presence of stage B heart failure. (a) Adjusted odds ratios (ORs) and 95% confidence intervals (CIs) for stage B heart failure according to the number of risk factors (0, 1, or 2), with the 0-factor group as reference. Compared to patients with low CAVI and low BNP (0 factors), those with one high parameter (one factor) showed a 3.81-fold higher odds (OR: 3.81; 95% CI: 1.54–9.41, *P = 0.004), and those with both high parameters (two factors) showed a 9.72-fold higher odds (OR: 9.72; 95% CI: 3.24–19.92, P < 0.001). (b) Comparison of the adjusted OR between subjects with both risk factors (two factors) and those with only one risk factor (one factor) as the reference. The adjusted OR for the two-factor group was 5.36 (95% CI: 2.39–12.90, P < 0.001). Risk factors are defined as CAVI > 9.0 and BNP > 34.0 pg/mL. Adjustment factor: calf circumference, RAS inhibitors, E/e' ratio, skin autofluorescence, SEVR, GLP-1 receptor agonists, age, eGFR, CAD, SGLT2 inhibitors. Data expressed OR and 95% CI. HF: heart failure; CAVI: cardio-ankle vascular index; BNP: brain natriuretic peptide; OR: odds ratio; CI: confidence interval; RAS: renin–angiotensin system; SEVR: subendocardial viability ratio; GLP-1: glucagon-like peptide-1; eGFR: estimated glomerular filtration rate; CAD: coronary artery disease; SGLT2: sodium glucose cotransporter 2.

The statistical analysis was conducted using the commercialized MedCalc software (version 22.0; MedCalc Software Ltd, Ostend, Belgium). Continuous variables are expressed as mean values and standard deviations or medians (interquartile ranges). Coefficients of the correlations between CAVI and various clinical parameters were expressed using Pearson’s r or Spearman’s ρ analysis. The correlation between CAVI and clinical characteristics was evaluated using Pearson’s correlation coefficient for normally distributed continuous variables (e.g., age, body mass index, echocardiographic parameters, and skin autofluorescence). For non-normally distributed continuous variables (BNP and hs-CRP) and categorical variables, Spearman’s rank correlation coefficient was applied. A multiple linear regression analysis was performed to identify factors independently associated with CAVI (Table 1). In addition, a multivariable logistic regression analysis was conducted to calculate the odds ratios (ORs) for stage B heart failure based on the combination of CAVI and BNP categories (Fig. 1). The 18 variables that had statistically significant associations with CAVI in the univariate analysis (age, HF stage, obesity, current smoker, CAD, interventricular septal thickness at end-diastole, left arterial dimension, E/e' ratio, hemoglobin A1c, skin autofluorescence, BNP, eGFR, hs-CRP, SEVR, calf circumference, sodium glucose cotransporter 2 (SGLT2) inhibitors use, glucagon-like peptide-1 (GLP-1) receptor agonists use, and RAS inhibitors use) were used as explanatory variables in the multivariable logistic regression analysis. Multicollinearity among independent variables was assessed and ruled out. In addition, the validity of the regression model was confirmed by a residual analysis, which demonstrated that the residuals followed a normal distribution. The optimal cut-off values of CAVI and BNP to distinguish stage A HF and B HF were decided via the receiver operating characteristic (ROC) curve analysis using the Youden index [22]. DeLong test was used as the comparison statistic to evaluate the difference between the two ROC curves. The threshold for statistical significance was set at P < 0.05. To address potential over-adjustment, a sensitivity analysis was performed using a simplified model adjusted only for age, sex, and traditional cardiovascular risk factors.

**Table 1:** Multivariate Analysis for CAVI

Variable	β	P value
Calf circumference	−0.21	< 0.001
RAS inhibitors	−0.19	< 0.001
E/e'	0.18	< 0.001
Skin autofluorescence	0.17	0.001
SEVR	−0.17	0.001
GLP-1 receptor agonists	−0.16	0.002
Age	0.15	0.003
eGFR	−0.14	0.004
CAD	0.13	0.012
Stage B HF (vs. stage A HF)	0.12	0.013
SGLT2 inhibitors	−0.11	0.014
LAD	0.08	0.067
HbA1c	0.08	0.071
Body mass index	−0.07	0.110
hs-CRP	0.06	0.203
BNP	0.05	0.377
IVSTd	0.04	0.496
Current smoker	0.02	0.623

R^2^ = 0.663, P < 0.001, β: standardized regression coefficient. RAS: renin-angiotensin system; SEVR: subendocardial viability ratio; GLP-1: glucagon-like peptide-1; eGFR: estimated glomerular filtration rate; CAD: coronary artery disease; HF: heart failure; hs-CRP: high-sensitivity C-reactive protein; LAD: left atrial dimension; HbA1c: hemoglobin A1c; SGLT2: sodium glucose cotransporter 2; BNP: brain natriuretic peptide; IVSTd: interventricular septal thickness at end-diastole.

## Results

### Baseline clinical characteristics

Table 2 indicates the baseline clinical characteristics of the study participants. The CAVI ranged from 5.2 to 14.1, with an average value of 9.2. Regarding the HF stage, 157 patients (54%) were in stage A HF, and 135 patients (46%) were in stage B HF. The average HbA1c level was 7.1%. In total, 175 patients (60%) were taking RAS inhibitors. However, regarding anti-diabetes medications, 214 patients (73%) were taking dipeptidyl peptidase 4 inhibitors, while SGLT2 inhibitors use and GLP-1 receptor agonists use were found in 78 patients (27%) and 23 patients (8%), respectively.

**Table 2:** Baseline Characteristics of the Study Population

Characteristics	N = 292
Age (years)	70 ± 7
Sex (men/women)	113/179
CAVI	9.2 ± 1.7
Stage (A HF/B HF)	157/135
Height (m)	1.59 ± 0.08
Body weight (kg)	59.3 ± 10.2
Body mass index (kg/m^2^)	23.3 ± 3.5
Current smoker, n (%)	39 (13)
CAD, n (%)	36 (12)
Systolic blood pressure (mm Hg)	134 ± 13
LDL cholesterol (mg/dL)	127 ± 39
HDL cholesterol (mg/dL)	49 ± 15
IVSTd (mm)	9.4 ± 2.0
LVDd (mm)	49.9 ± 3.4
LVEF (%)	62.6 ± 8.1
LAD (mm)	43.3 ± 6.1
E/e'	8.4 ± 2.2
Fasting blood glucose (mg/dL)	143 ± 26
HbA1c (%)	7.1 ± 1.0
Skin autofluorescence (AU)	2.7 ± 0.6
BNP (pg/mL)	29.0 (22.0–64.0)
eGFR (mL/min/1.73 m^2^)	62.3 ± 16.5
hs-CRP (mg/L)	0.90 (0.31–2.33)
SEVR (%)	140 ± 24
Calf circumference (cm)	35.3 ± 4.7
Medication	
Metformin, n (%)	129 (44)
DPP-4 inhibitors, n (%)	214 (73)
SGLT2 inhibitors, n (%)	78 (27)
GLP-1 receptor agonists, n (%)	23 (8)
Insulin, n (%)	25 (9)
RAS inhibitors, n (%)	175 (60)
β blockers, n (%)	50 (17)
Statins, n (%)	150 (51)

Continuous values are mean ± SD or median (25th–75th percentile). CAVI: cardio-ankle vascular index; HF: heart failure; CAD: coronary artery disease; LDL: low-density lipoprotein; HDL: high-density lipoprotein; IVSTd: interventricular septal thickness at end-diastole; LVDd: left ventricular end-diastolic diameter; LVEF: left ventricular ejection fraction; LAD: left atrial dimension; HbA1c: hemoglobin A1c; AU: arbitrary unit; BNP: brain natriuretic peptide; eGFR: estimated glomerular filtration rate; hs-CRP: high-sensitivity C-reactive protein; SEVR: subendocardial viability ratio; DPP-4: dipeptidyl peptidase 4; SGLT2: sodium glucose cotransporter 2; GLP-1: glucagon-like peptide-1; RAS: renin–angiotensin system.

### Correlations between CAVI and various clinical parameters

Table 3 presents the correlations between CAVI and various clinical parameters. CAVI had significantly positive correlations with age, stage B HF, current smoking, history of CAD, interventricular septal thickness at end-diastole, left atrial dimension, E/e' ratio, hemoglobin A1c, skin autofluorescence, BNP, and hs-CRP. However, it had significantly negative correlations with obesity, eGFR, SEVR, calf circumference, RAS inhibitors use, SGLT2 inhibitors use, and GLP-1 receptor agonists use.

**Table 3:** Correlation Between CAVI and Various Clinical Characteristics

Characteristics	Correlation coefficient	P value
Age (actual value)	0.30	< 0.001
Sex (women = 0, men = 1)	0.03	0.608
Stage (A HF = 0, B HF = 1)	0.31	< 0.001
Body mass index (actual value)	−0.21	0.001
Current smoker (no = 0, yes = 1)	0.18	0.002
CAD (no = 0, yes = 1)	0.19	0.001
Systolic blood pressure (actual value)	0.09	0.107
LDL cholesterol (actual value)	−0.04	0.502
HDL cholesterol (actual value)	−0.08	0.156
IVSTd (actual value)	0.12	0.048
LVDd (actual value)	0.11	0.060
LVEF (actual value)	0.09	0.109
LAD (actual value)	0.25	< 0.001
E/e' (actual value)	0.39	< 0.001
Fasting blood glucose (actual value)	0.09	0.119
HbA1c (actual value)	0.23	< 0.001
Skin autofluorescence (actual value)	0.43	< 0.001
BNP (actual value)	0.26	< 0.001
eGFR (actual value)	−0.35	< 0.001
hs-CRP (actual value)	0.20	0.006
SEVR (actual value)	−0.49	< 0.001
Calf circumference (actual value)	−0.33	< 0.001
Metformin (no = 0, yes = 1)	−0.03	0.583
DPP-4 inhibitors (no = 0, yes = 1)	−0.11	0.063
SGLT2 inhibitors (no = 0, yes = 1)	−0.25	< 0.001
GLP-1 receptor agonists (no = 0, yes = 1)	−0.26	< 0.001
Insulin (no = 0, yes = 1)	0.06	0.349
RAS inhibitors (no = 0, yes = 1)	−0.26	< 0.001
β blockers (no = 0, yes = 1)	0.06	0.327
Statins (no = 0, yes = 1)	−0.04	0.490

Data are expressed as Pearson’s r or Spearman’s ρ. CAVI: cardio-ankle vascular index; HF: heart failure; CAD: coronary artery disease; LDL: low-density lipoprotein; HDL: high-density lipoprotein; IVSTd: interventricular septal thickness at end-diastole; LVDd: left ventricular end-diastolic diameter; LVEF: left ventricular ejection fraction; LAD: left atrial dimension; HbA1c: hemoglobin A1c; BNP: brain natriuretic peptide; eGFR: estimated glomerular filtration rate; hs-CRP: high-sensitivity C-reactive protein; SEVR: subendocardial viability ratio; DPP-4: dipeptidyl peptidase 4; SGLT2: sodium glucose cotransporter 2; GLP-1: glucagon-like peptide-1; RAS: renin–angiotensin system.

### Multiple regression analysis

Table 1 presents the results of the multivariable regression analysis for CAVI. Of the 18 explanatory variables associated with CAVI by simple correlation, 11 explanatory factors (calf circumference, RAS inhibitors use, E/e' ratio, skin autofluorescence, SEVR, GLP-1 receptor agonists use, age, eGFR, CAD, stage B HF, and SGLT2 inhibitors use) had independent associations with CAVI (R^2^ = 0.663). However, BNP was not selected as independent variables for CAVI as dependent variable.

### CAVI threshold of the combination of CAVI and BNP for distinguishing between stage A HF and stage B HF

**Figure 2:**
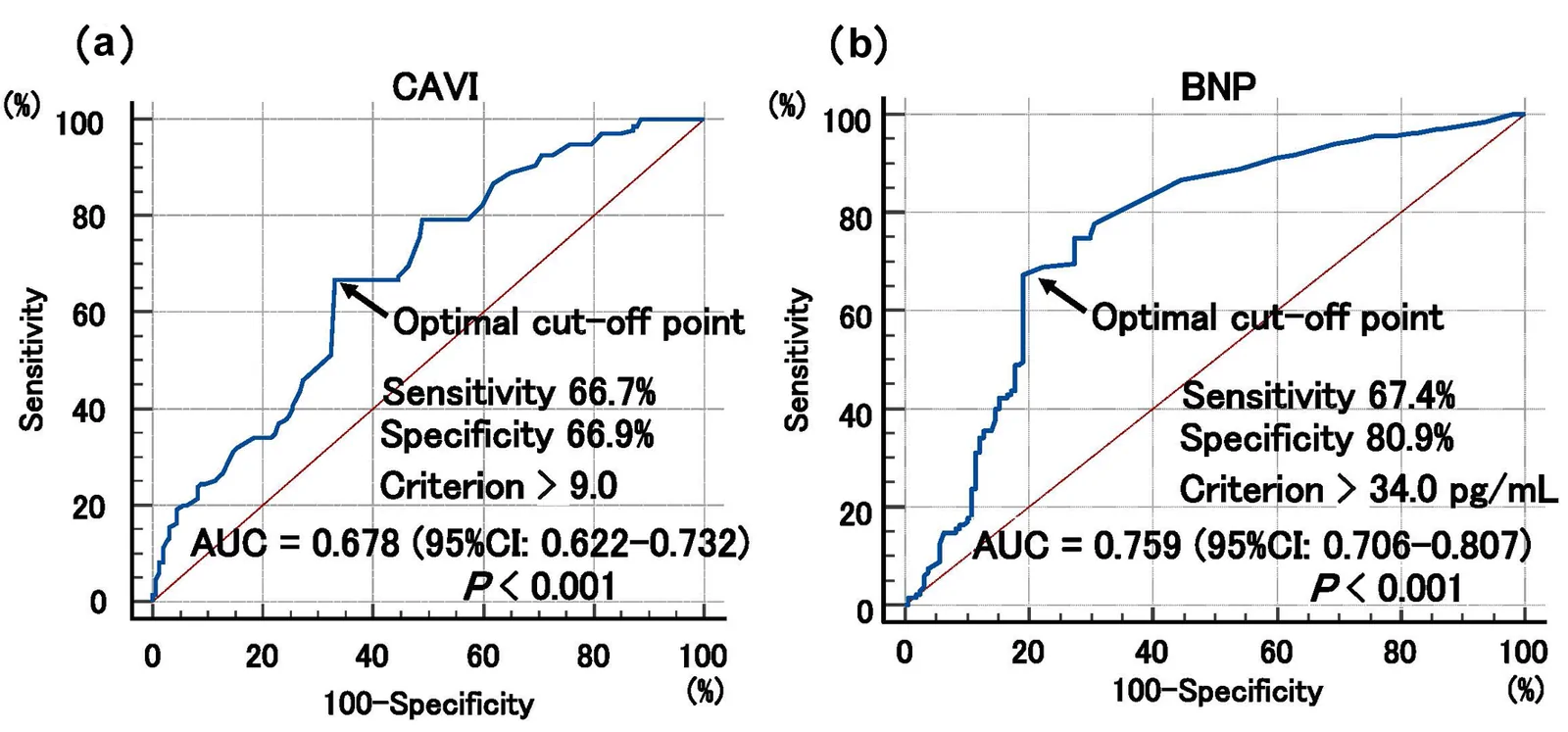
The optimal cut-off values of CAVI and BNP threshold for distinguishing between stage A HF and stage B HF using the receiver operating characteristic curve analysis. This analysis indicated that 9.0 is the CAVI threshold for distinguishing between the two HF stages (a). For BNP, 34.0 pg/mL was the threshold for distinguishing between the two HF stages (b). Comparison between the AUC of CAVI and BNP was performed using the DeLong test (P = 0.073). CAVI: cardio-ankle vascular index; BNP: brain natriuretic peptide; HF: heart failure; AUC: area under the curve; CI: confidence interval.

Figure 2 presents CAVI and BNP thresholds for distinguishing between stage A HF and stage B HF via the ROC curve analysis using the Youden index. This analysis indicated that 9.0 is the CAVI threshold for distinguishing between the two HF stages (Fig. 2a). For BNP, 34.0 pg/mL was the threshold for distinguishing between the two HF stages (Fig. 2b). Figure 1 indicates the combination of CAVI and BNP to distinguish stage A HF and stage B HF using multiple logistic regression analysis. Compared to patients with low CAVI and low BNP, patients for whom one of these parameters was high showed a 3.81-fold higher odds (OR: 3.81; 95% confidence interval (CI): 1.54–9.41, P = 0.004), and those in whom both parameters were high showed 9.72-fold higher odds (OR: 9.72; 95% confidence interval: 3.24–19.92, P < 0.001, Fig. 1a). In the comparison of the adjusted OR between subjects with both risk factors (two factors) and those with only one risk factor (one factor) as the reference, the adjusted OR for the two-factor group was 5.36 (95% CI: 2.39–12.90, P < 0.001, Fig. 1b).

## Discussion

This study examined the clinical significance of CAVI, an indicator of arterial stiffness, in stage A/B HF patients with T2DM. Although CAVI was associated with various clinical parameters, the multiple regression analysis revealed independent associations with 11 factors by high levels of the multiple correlation coefficient, as R^2^ = 0.663. Of these, the associations between age and CAD and CAVI were consistent with previous reports [23–25]. Regarding the relationship between cardiac function indicators and CAVI, independent associations were found with E/e' (an indicator of diastolic function) and SEVR (a representation of subendocardial ischemia). The importance of AGEs was demonstrated in the association between blood glucose-related factors and CAVI. Furthermore, an independent association was found with decrease in eGFR (an indicator of renal function), suggesting that CAVI is an important factor connecting the cardio-renal interaction in stage A/B HF patients with T2DM. In addition, calf circumference, an indicator of sarcopenia, was selected as the strongest explanatory factor for CAVI in this study. Regarding medications, independent associations were found between CAVI and novel anti-diabetes medications, such as SGLT2 inhibitors and GLP-1 receptor agonists, in addition to RAS inhibitors. However, another important finding obtained in this study was the usefulness of evaluating CAVI and BNP together in distinguishing between stage A HF and stage B HF in patients with T2DM.

### Relationships among left ventricular diastolic dysfunction, subendocardial ischemia, and CAVI

In recent years, the importance of left ventricular diastolic function in cases with preserved left ventricular systolic function has been demonstrated in HF pathogenesis. However, the mean LVEF in the study group was 62.6%, suggesting that most cases had preserved left ventricular systolic function, highlighting the importance of left ventricular diastolic function in this study group. In addition, there have been several reports showing a correlation between elevated arterial stiffness and left ventricular diastolic function, and several reports have shown a correlation between CAVI and markers of diastolic function, including the E/e' ratio [26, 27], indicating that the importance of increase in CAVI as a marker of arterial stiffness for diastolic dysfunction. This may be explained by the ventricular-vascular coupling mechanism, which demonstrates how increased arterial stiffness (high CAVI) elevates left ventricular afterload and alters myocardial properties, which ultimately leads to left ventricular diastolic dysfunction and a subsequent increase in left ventricular filling pressure (high E/e'). In addition, the results of this study support the validity of existing reports, and the present findings suggest that monitoring CAVI from an early stage may help identify patients with subclinical diastolic dysfunction. However, due to the cross-sectional design of this study, it remains unclear whether maintaining a lower CAVI can directly prevent the progression of diastolic dysfunction in stage A/B HF patients with T2DM. As mentioned above, SEVR is a finding indicating subendocardial ischemia, and, in recent years, there have been reports that decreased SEVR is associated with HF progression [28, 29]. Furthermore, Han et al demonstrated that arterial stiffness is associated with decreased SEVR [30]. This study also demonstrated an independent involvement of CAVI elevation in the decrease in SEVR in stage A/B HF patients with T2DM, suggesting that preventing an increase in CAVI is important from the perspective of subendocardial ischemia. However, due to its cross-sectional design, this study could not determine the incidence of HF; therefore, further prospective studies are required to evaluate reductions in HF incidence by increased SEVR through CAVI improvement in patients with T2DM.

### AGEs, arterial stiffness, and HF

AGEs are deeply involved in various pathophysiological aspects in systemic organs of patients with T2DM, and several studies have indicated that AGEs and AGEs receptors are involved in HF pathogenesis [31–33]. In addition, existing reports have indicated that skin autofluorescence is involved in HF pathogenesis [34–36]. However, a significant association has been reported between skin autofluorescence and arterial stiffness including CAVI [37, 38], suggesting that AGEs act on vascular stiffness through the pathway of inflammation and oxidative stress in vascular wall. In this study as well, among the blood glucose-related factors, only skin autofluorescence was identified as an independent determinant of CAVI, an indicator of arterial stiffness in stage A/B HF patients with T2DM. This indicates a close association between AGEs and arterial stiffness, as reflected in CAVI, in stage A/B HF patients with T2DM. Although these findings do not prove a causal relationship or that treating AGEs will prevent the development of symptomatic HF, they highlight a potential pathophysiological link that warrants further prospective investigation.

### Sarcopenia and CAVI

Several basic and clinical studies have highlighted the importance of sarcopenia in HF pathogenesis, with inflammation, oxidative stress, and sympathetic nervous system activation being cited as contributing factors [39, 40]. In addition, several researchers have reported an association between sarcopenia and vascular function, inferring the pathogenesis of HF from a decline in vascular function [41, 42]. Meanwhile, several previous studies have shown an association between CAVI and sarcopenia [43–45], supporting the existence of a close relationship between the two. In this study as well, calf circumference, an indicator of sarcopenia, was selected as the strongest contributing factor to CAVI, even though reliability of calf circumference as a marker of sarcopenia. Therefore, considering existing reports alongside the results of this study, the findings demonstrate a significant statistical association between sarcopenia and vascular dysfunction (including an increase in CAVI) as interrelated features in stage A/B HF patients with T2DM. This association may be mediated by shared biological mechanisms, such as chronic inflammation, oxidative stress, and altered adipokine profiles. While active detection of sarcopenia may be useful for comprehensive risk stratification, it should be noted that calf circumference primarily reflects muscle quantity. A definitive diagnosis of sarcopenia requires a comprehensive assessment of muscle quality, including muscle strength and physical function. Furthermore, longitudinal studies are necessary to elucidate causal direction and to confirm whether clinical interventions for sarcopenia can actually reduce the future incidence of HF in this population.

### Medications and CAVI

RAS inhibitors are known to be involved in various pathological conditions associated with T2DM, and their use is recommended for patients with this metabolic disease. However, several researchers have reported that RAS inhibitors reduce CAVI [46, 47], and this study’s findings are consistent with existing reports. However, the effect of RAS inhibitors on HF with preserved ejection fraction is limited [48, 49]. Since the study group mainly consisted of cases with preserved LVEF, it is likely that the effect of RAS inhibitors on HF prevention is limited. Nevertheless, it is expected that RAS inhibitor use in patients with high CAVI may efficiently lead to the development of HF. To confirm this in the future, the usefulness of RAS inhibitors in preventing HF in cases with preserved LVEF should be investigated from the perspective of CAVI. Recently, SGLT2 inhibitors and GLP-1 receptor agonists have attracted attention as novel T2DM treatment options. Both medications are attracting attention for their additional benefit of acting on the cardiovascular system [50, 51], and an independent relationship between both medications and CAVI was observed in this cross-sectional study. Sakai et al reported that SGLT2 inhibitors such as empagliflozin, luseogliflozin, and tofogliflozin lower CAVI [52]. However, to the best of the author’s knowledge, there are no reports showing an association between GLP-1 receptor agonists and CAVI. Nevertheless, a negative correlation between CAVI and GLP-1 receptor agonists was observed in the multivariate analysis of this study. Several reports describe the mechanisms by which GLP-1 receptor agonists affect vascular function such as vascular fibrosis, endothelial dysfunction, inflammation [53, 54], and oxidative stress, and these underlying pathways might support a potential link with arterial stiffness. In addition, despite its cross-sectional design, the results of this study suggest a potential association between GLP-1 receptor agonist use and lower CAVI. Hence, as detailed in the “Limitations” section, prospective interventional studies are mandatory to confirm whether this reflects a true medication effect. Furthermore, because recent clinical evidence suggests that SGLT2 inhibitors and GLP-1 receptor agonists may potentially reduce skeletal muscle mass or lean body mass, clinicians should be cautious regarding the risk of sarcopenia progression [55, 56]. Consequently, the implementation of appropriate protein intake and structured exercise regimens is highly recommended for patients with T2DM when prescribing these two classes of medications.

### Combination of CAVI and BNP to distinguish stage A HF and stage B HF

In this study, a significant positive correlation was observed between CAVI and BNP in a simple correlation analysis, although this association was not statistically significant in the multivariate analysis. This suggests that CAVI reflects pathological conditions involved in HF progression that cannot be captured by BNP alone in early-stage HF. Therefore, the author first determined cut-off values for distinguishing between stage A HF and stage B HF from the ROC curve analysis. As a result, the author calculated cut-off values of 9.0 for CAVI and 34 pg/mL for BNP. A CAVI of 9 was consistent with existing reports that, although the target groups differ, CAVI is generally considered a risk factor for CVD [16, 57, 58]. However, several recent reports have used BNP values of ≥ 35 pg/mL to distinguish between stage A HF and stage B HF, which is similar to the value in this study [59, 60]. Therefore, the findings support the validity of the current guideline cutoff values for BNP levels in patients with T2DM in routine clinical practice. Next, using these thresholds, the author performed a multiple logistic regression analysis. As the number of factors (high CAVI, high BNP) increased, the OR increased compared with lower levels of both parameters. Needless to say, numerous factors are involved in the progression of HF, and it is difficult to completely distinguish between stage A and stage B HF using only two markers as CAVI and BNP. However, the results of this study suggest that there are cases in which progression from stage A to stage B HF can be prevented by combining CAVI and BNP. Although the multiple regression in Figure 1 carried a risk of over-adjustment, a simplified sensitivity analysis adjusting only for core demographic factors yielded consistent and robust ORs. While the prospective multicenter study by Miyoshi et al [18] (previously cited in the “Introduction”) demonstrated the long-term prognostic value of CAVI for future cardiovascular outcomes, the present study uniquely adds to the literature by focusing strictly on a highly selected population of patients with T2DM. Furthermore, this study demonstrates the immediate clinical utility of combining CAVI with BNP levels to specifically stratify the risk of subclinical progression from stage A to stage B HF. This combined approach provides primary care clinicians with an actionable screening strategy within routine diabetic management, complementary to the broader prognostic framework established by Miyoshi et al. A notable methodological strength of the present study lies in the group assignment criteria for stage A and B HF. The author intentionally relied strictly on structural and clinical parameters—rather than BNP levels—for the initial classification. By doing so, the author successfully avoided circular reasoning when evaluating the subsequent combined utility of CAVI and BNP. Consequently, the remarkably high OR (9.72-fold) observed in patients with both high CAVI and high BNP represents a clinically objective and robust finding, free from selection bias.

### Limitations

This study has several noteworthy limitations. First, it was conducted only on East Asian stage A/B HF patients with T2DM, focusing on a relatively small number of cases at a single facility. Therefore, future larger-scale, multicenter studies are anticipated to include other ethnicities to produce more generalizable findings. Second, the observed association between medication use, particularly GLP-1 receptor agonists, and lower CAVI values must be interpreted with caution. These findings are subject to confounding by indication inherent in non-randomized, cross-sectional designs. Given that GLP-1 receptor agonists were used by only 23 participants (8% of the cohort), this specific finding should be considered purely hypothesis-generating and requires validation in large-scale randomized controlled trial. Future large-scale, multicenter studies with a larger sample size of GLP-1 receptor agonists users are needed to confirm these findings. Third, the diagnostic accuracy of CAVI as a standalone screening tool for distinguishing between stage A and stage B HF was modest, as demonstrated by an AUC of 0.678, with a sensitivity of 66.7% and a specificity of 66.9%. Therefore, the threshold of ≤ 9.0 identified in the present study must be interpreted strictly as an exploratory cut-point requiring future external validation, rather than an established therapeutic target. Additionally, it should be noted that the ROC-derived optimal BNP cut-off value of 34.0 pg/mL sits slightly above the median BNP level of 29.0 pg/mL for the entire study population. This baseline distribution may limit the immediate generalizability of this specific threshold to other cohorts with different background demographics, although it represents a clinically practical screening value for the present study population. Fourth, due to the relatively small sample size and cross-sectional design of this single-center study, the potential for residual confounding from other unmeasured disease processes cannot be fully eliminated. Consequently, while the independent statistical association of CAVI and BNP suggests they may reflect distinct pathophysiological pathways (macrovascular stiffness versus myocardial wall stress), concluding that their combination provides superior clinical risk stratification remains a hypothesis that requires validation. Prospective, large-scale longitudinal studies are mandatory to confirm the clinical utility of this dual-marker approach in routine practice. Finally, while this study yielded some important insights into the significance of CAVI in stage A/B HF patients with T2DM, some questions remain. Therefore, further detailed research from both clinical and basic research perspectives is needed to clarify the significance of CAVI in stage A/B HF patients with T2DM. In addition, the validity of the results of this study needs to be verified through prospective trials. However, this study focused solely on the association between CAVI and stage A/B HF. Therefore, further research is needed regarding the association between CAVI and other diabetic complications, particularly those in the early stages.

### Conclusions

This study suggests that CAVI is closely associated with pathological indicators in stage A/B HF patients with T2DM. Furthermore, the findings indicate that a combination of CAVI and BNP measurements could serve as a potential screening tool for risk stratification in the pre-symptomatic stage. Given the cross-sectional nature of this study, caution is warranted in inferring causality, and the author cannot conclude that therapeutic interventions targeting CAVI directly prevent HF progression; nevertheless, this combination holds potential for early risk stratification. To confirm these findings and improve generalizability, future large-scale, prospective multicenter clinical trials are warranted.

## Data Availability

The author declares that data supporting the findings of this study are available within the article.

## Abbreviations

AGEs: advanced glycation end products
AU: arbitrary unit
AUC: area under the curve
BNP: brain natriuretic peptide
CAVI: cardio-ankle vascular index
CAD: coronary artery disease
CVD: cardiovascular disease
eGFR: estimated glomerular filtration rate
GLP-1: glucagon-like peptide-1
HF: heart failure
LVEF: left ventricular ejection fraction
OR: odds ratio
RAS: renin–angiotensin system
SEVR: subendocardial viability ratio
SGLT2: sodium glucose cotransporter 2
T2DM: type 2 diabetes mellitus
